# The safety and efficacy of tPA intravenous thrombolysis for treating acute ischemic stroke patients with a history of cerebral hemorrhage

**DOI:** 10.1590/1414-431X20187739

**Published:** 2019-01-24

**Authors:** Guang-jian Zhao, Zi-ran Wang, Fan-zhen Lin, Yan-sen Cui, Shun-liang Xu

**Affiliations:** 1 Department of Neurology, Linyi People's Hospital Affiliated to Shandong University, Linyi, Shandong, China; 2 General Medicine, Linyi People's Hospital Affiliated to Shandong University, Linyi, Shandong, China; 3 Department of Neurology, The Second Hospital of Shandong University, Jinan, Shandong, China

**Keywords:** tPA, Acute ischemic stroke, History of cerebral hemorrhage, Intravenous thrombolysis, Contraindication

## Abstract

Alteplase (tPA) intravenous thrombolysis is an effective treatment for acute ischemic stroke (AIS) when administered within 4.5 h of initial stroke symptoms. Here, its safety and efficacy were evaluated among AIS patients with a previous history of cerebral hemorrhage. Patients who arrived at the hospital within 4.5 h of initial stroke symptoms and who were treated with tPA intravenous thrombolysis or conventional therapies were analyzed. The 90-day modified Rankin scale (90-d mRS) was used alongside mortality and incidence of symptomatic intracerebral hemorrhage (SICH) rates to evaluate the curative effect of these therapies. Among 1,694 AIS patients, 805 patients were treated with intravenous thrombolysis, including patients with (n=793) or without (n=12) a history of cerebral hemorrhage, and the rate of incidence of SICH significantly differed between them (8.3 *vs* 4.3%, P=0.039). No significant difference was found in 90-d mRS measurements (41.7 *vs* 43.6%, P=0.530) and 90-d mortality rates (8.3 *vs* 6.5%, P=0.946). A total of 76 AIS patients with a history of cerebral hemorrhage received tPA thrombolytic therapy (n=12) or conventional therapy (n=64), and a significant difference was noted in the 90-d mRS scores between the two groups (41.7 *vs* 23.4%, P=0.029), while no significant difference was found in SICH measurements (8.3 *vs* 4.6%, P=0.610) and 90-d mortality rates (8.3 *vs* 9.4%, P=0.227). A history of cerebral hemorrhage is not an absolute contraindication for thrombolytic therapy; tPA intravenous thrombolysis does not increase SICH measurements and mortality rates in patients with a history of cerebral hemorrhage, and they may benefit from thrombolytic therapy.

## Introduction

Acute ischemic stroke (AIS) is a major cause of death and disability in the United States (1). Hypertension is a common risk factor for both ischemic and hemorrhagic strokes (2), and incidence of hypertensive intracerebral hemorrhage in the Asian population is higher than in Europe and the United States due to racial and dietary habits (3). The use of alteplase (tPA) intravenous thrombolysis within 4.5 h of symptom onset during stroke is the only therapy that reduces disability among AIS patients (4).

Early clinical trials of intravenous thrombolytic therapy for AIS have primarily been designed to avoid increased symptomatic intracerebral hemorrhage (SICH), mortality rates, and negative treatment outcomes (5). Currently, the stroke guidelines of every country classify a history of intracranial hemorrhage as a contraindication for intravenous thrombolytic therapy (6,7). Thus, patients with a history of cerebral hemorrhage cannot receive intravenous thrombolysis, regardless of the location and amount of bleeding or recovery situation.

Contraindication in stroke guidelines is based on contraindications from previous randomized controlled clinical trials, which lack rigorous clinical evidence (8). Recent studies have shown that contraindications for intravenous thrombolysis are too stringent and that some patients do not receive effective treatment for stroke because of this. In fact, the use of intravenous thrombolysis for AIS is supported by an increasing number of studies (9,10), while contraindications continue to be shown as unnecessary through clinical experience (11). However, the ability of AIS patients with a history of cerebral hemorrhage to accept tPA thrombolytic therapy remains in question, and the safety of this treatment requires further evaluation. Thus, the present study evaluated the safety and efficacy of intravenous thrombolysis for AIS patients with a previous history of cerebral hemorrhage.

## Material and Methods

### Patients

This retrospective study was approved by the Ethics Committee of the Linyi People's Hospital Affiliated to Shandong University and followed the ethical principles stated in the Declaration of Helsinki and the relevant laws of the People's Republic of China. Written informed consent was obtained from all patients.

The study retrospectively analyzed patients with AIS who were treated in Linyi People's Hospital Affiliated to Shandong University from July 2015 to December 2017. The use rate of tPA (Boehringer Ingelheim, GmbH) intravenous thrombolytic therapy and the treatment situations of AIS patients with previous histories of cerebral hemorrhage within 4.5 h of arrival at the hospital were obtained. Patients who underwent bridge therapy were not included in this study. All patients treated with tPA thrombolysis had undergone laboratory tests, head imaging, and were measured according to the National Institutes of Health Stroke Scale (NIHSS) before thrombolysis. Within 24 h of tPA thrombolysis treatment, all patients received necessary nursing supervision, such as electrocardiograph monitoring. When cerebral hemorrhage was controlled at 24 h, patients received antithrombotic treatment and other symptomatic treatments for certain risk factors. Follow-up head imaging was performed 24–36 h after the onset of stroke, followed by NIHSS scoring, and a modified Rankin scale (mRS) score was taken with a 90-day (±7 d) follow-up after the onset of stroke.

### Patients with a history of cerebral hemorrhage

History of cerebral hemorrhage is defined as a definite history of spontaneous cerebral hemorrhage confirmed by previous cerebral computed tomography. However, subdural hematomas caused by trauma and subarachnoid hemorrhages were excluded from this study. For safety, patients with a history of cerebral hemorrhage were treated with a low dose of tPA (0.6 mg/kg).

### Evaluation of safety and effectiveness

The main safety outcome measures were SICH after thrombolysis and the mortality rate of patients at 90 days after the onset of stroke. Based on the research standards of the European Cooperative Acute Stroke Study II (9), SICH is defined as bleeding indicated by head image examination after thrombolytic therapy and a NIHSS score of 4 points or more than the baseline NIHSS score or than the baseline NIHSS score for 24–36 h after thrombolysis. The primary effective outcome was measured using the 90-d mRS score after thrombolysis (10), with a score of 0–1 indicating a good clinical outcome, 2–5 indicating varying degrees of dysfunction, and 6 indicating death.

### Statistical analysis

The SPSS 22.0 software package was used for statistical analysis. The measurement data are reported as a median and four quantile intervals, and the difference between groups was examined using a Mann-Whitney U test. Count data are reported as frequency and percentage, and a χ^2^ test was used to analyze the difference between the two treatment groups. The odds ratio (OR) and the 95% confidence interval (CI) for safety and effectiveness outcomes and the modified OR and its 95%CI were compared by univariate and multivariate regression analyses, with P<0.05 being statistically significant.

## Results

### General data and baseline characteristics

**Figure 1.**
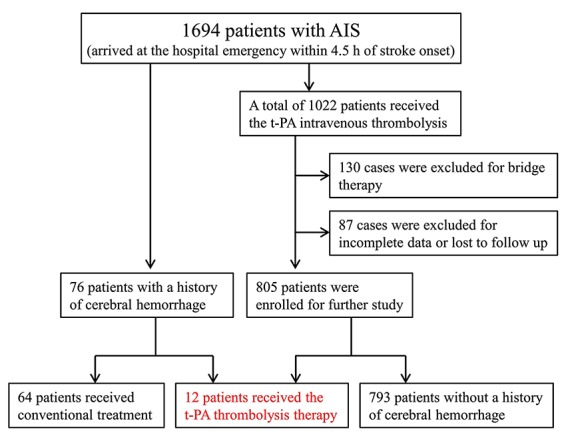
Patient flowchart: Among 1,694 acute ischemic stroke (AIS) patients, 805 patients received the alteplase (t-PA) intravenous thrombolysis were enrolled in the study, including 793 patients without a history of cerebral hemorrhage. In addition, 76 AIS patients had a history of cerebral hemorrhage, including 12 patients who received tPA thrombolytic therapy and 64 patients who received conventional treatment.

The study was a retrospective analysis of 17,285 patients with AIS admitted to Linyi People's Hospital from July 2015 to December 2017, with 1,694 patients arriving at the hospital emergency department within 4.5 h of the onset of stroke. Among these patients, 1,022 received t-PA intravenous thrombolysis, and 130 cases were excluded due to bridge therapy and 87 cases due to incomplete data or lost to follow-up. Thus, 805 patients with AIS received the t-PA intravenous thrombolysis were enrolled in this study, including 793 patients without a previous history of cerebral hemorrhage. There were 76 AIS patients with a history of cerebral hemorrhage, including 12 patients who received tPA thrombolytic therapy, and 64 patients who received conventional treatments (Figure 1).

Among the patients who received tPA intravenous thrombolytic therapy, differences in patients who received a median tPA dose (0.6 *vs* 0.79 mg/kg, P=0.003) and who had a history of hypertension (83.3 *vs* 62.0%, P=0.028) and drinking (50 *vs* 30.5%, P=0.039) were significant for patients with and without a history of cerebral hemorrhage. There was no significant difference in other baseline characteristics, such as age, gender, NIHSS score, mRS score of 0–1 before stroke, history of diabetes mellitus, or atrial fibrillation (Table 1).

**Table 1.** Demographic and baseline clinical data for patients undergoing intravenous thrombolysis.

Variables	With previousintracerebral hemorrhage(n=12)	Without previous intracerebral hemorrhage (n=793)	P value
Demographic factors			
Age (year; M, IQR)	66 (60–73)	66 (60–72)	0.316
Male (n, %)	7 (58.3)	490 (61.8)	0.835
Median tPA dose (mg/kg)	0.64 (0.60–0.69)	0.79 (0.60–0.90)	0.003
Before stroke mRS 0-1scores (n, %)	11 (91.7)	737 (94.5)	0.611
Vascular risk factors (n, %)			
Hypertension	10 (83.3)	492 (62.0)	0.028
Hyperlipidemia	4 (33.3)	301 (37.9)	0.837
Diabetes	2 (16.7)	153 (19.3)	0.561
Ischemic heart disease	5 (41.7)	260 (32.8)	0.157
Atrial fibrillation	3 (25.0)	191 (24.1)	0.486
Smoking	5 (41.7)	350 (44.1)	0.302
Drinking	6 (50.0)	242 (30.5)	0.039
Stroke etiology typing (n, %)			
Atherosclerosis	5 (41.7)	305 (38.5)	0.809
Arteriolar occlusion	4 (33.3)	270 (34.0)	0.761
Cardiogenic embolism	2 (16.7)	176 (22.2)	0.297
Other determined causes	1 (8.3)	23 (2.9)	0.180
Undetermined etiology	0 (0)	19 (2.4)	1.0
Baseline NIHSS score (M, IQR)	13 (10–16)	12 (10–15)	0.483
Infarction located in the anterior circulation (n, %)	9 (75.0)	629 (79.3)	0.584

M: median; IQR: interquartile range; tPA: alteplase; mRS: modified Rankin scale; NIHSS: National Institutes of Health Stroke Scale. Statistical analysis was done with Mann-Whitney U test and χ^2^ test.

In patients with a previous history of cerebral hemorrhage, in addition to mRS scores of 0–1 before stroke (91.7 *vs* 78.1%, P=0.022), the time interval of cerebral hemorrhage occurrence (6.5 *vs* 3 years, P=0.003) was markedly different among patients who received tPA thrombolytic therapy or conventional therapy. There was no significant difference in other baseline features, including age, gender, NIHSS score, median tPA dose, and history of diabetes mellitus or atrial fibrillation (Table 2).

**Table 2.** Demographic and baseline clinical data of patients with previous intracerebral hemorrhage.

Variables	Intravenous thrombolysis (n=12)	Conventional therapy (n=64)	P value
Demographic factors			
Age (year; M, IQR)	66 (60–73)	67 (58–72)	0.185
Male (n, %)	7 (58.3)	39 (61.0)	0.291
Before stroke mRS (0-1) (n, %)	11 (91.7)	50 (78.1)	0.022
Vascular risk factors (n, %)			
Hypertension	10 (83.3)	53 (82.8)	0.625
Hyperlipidemia	4 (33.3)	24 (37.5)	0.334
Diabetes	2 (16.7)	13 (20.3)	0.513
Ischemic heart disease	5 (41.7)	25 (39.1)	0.610
Atrial fibrillation	3 (25.0)	15 (23.4)	0.495
Smoking	5 (41.7)	28 (43.8)	0.121
Drinking	6 (50.0)	33 (51.6)	0.364
Time interval of cerebral hemorrhage occurrence (year)	6.5 (5–8.75)	3 (2–6)	0.007
Stroke etiology typing (n, %)			
Atherosclerosis	5 (41.7)	27 (42.2)	0.156
Arteriolar occlusion	4 (33.3)	21 (32.8)	0.512
Cardiogenic embolism	2 (16.7)	13 (20.3)	0.249
Other determined causes	1 (8.3)	3 (4.7)	0.655
Undetermined etiology	0 (0)	1 (1.6)	1.0
Baseline NIHSS score (M, IQR)	13 (10–16)	14 (11–16)	0.196
Infarction located in the anterior circulation (n, %)	9 (75.0)	49 (76.5)	0.362

M: media; IQR: interquartile range; mRS: modified Rankin scale; NIHSS: National Institutes of Health Stroke Scale. Statistical analysis was done with Mann-Whitney U test and χ^2^ test.

### Safety and effectiveness of intravenous thrombolysis

**Figure 2.**
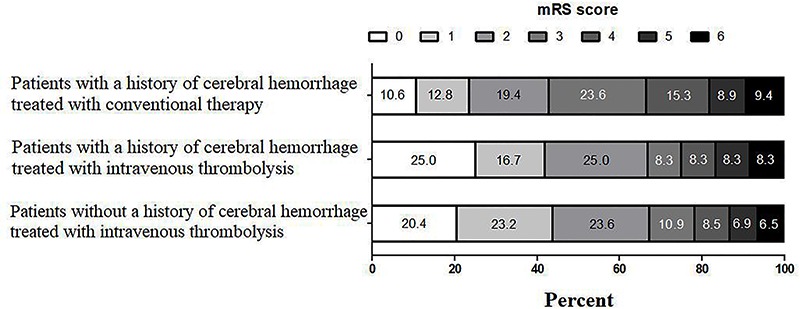
Functional outcomes at 90 days, according to modified Rankin scale (mRS) score (0–6): a score of 0 indicated no symptoms, 1 indicated symptoms without clinically significant disability, 2 indicated slight disability, 3 indicated moderate disability, 4 indicated moderately severe disability, 5 indicated severe disability, and 6 indicated death.

In patients who received tPA intravenous thrombolytic therapy, SICH (8.3 *vs* 4.3%, P=0.039) results were significantly different compared to results from patients with or without a history of cerebral hemorrhage, while no significant difference was observed for 90-d mRS scores of 0–1 (41.7 *vs* 43.6%, P=0.530) and 90-d mortality rate (8.3 *vs* 6.5%, P=0.946) (Table 3). In patients with a previous history of cerebral hemorrhage, a 90-d mRS score of 0–1 (41.7 *vs* 23.4%, P=0.029) was significantly different between those who received tPA thrombolytic therapy and those who received conventional therapy, although no significant difference was noted in SICH (8.3 *vs* 4.6%, P=0.610) and 90-d mortality rate (8.3 *vs* 9.4%, P=0.227) (Table 4). Figure 2 presents the mRS scores of the three groups and scores at the 90-d follow-up.

**Table 3.** Comparison of the efficacy and safety of intravenous thrombolysis in patients.

Outcomes	With previous intracerebral hemorrhage (n=12)	Without previous intracerebral hemorrhage (n=793)	OR (95%CI)	χ^2^	P value
90-d mRS score (0–1) (n, %)	5 (41.7)	346 (43.6)	0.539 (0.146–2.538)	0.527	0.530
Symptomatic cerebral hemorrhage	1 (8.3)	34 (4.3)	0.072 (0.0013–0.917)	3.912	0.048
90-d mortality (n, %)	1 (8.3)	52 (6.5)	0.837 (0.068–1.416)	0.028	0.946

OR: odds ratio; CI: confidence interval; mRS: modified Rankin scale.

**Table 4.** Comparison of the efficacy and safety of intravenous thrombolysis among patients with previous intracerebral hemorrhage.

Outcomes	Intravenous thrombolysis (n=12)	Conventional therapy (n=64)	OR (95%CI)	χ^2^	P value
90-d mRS score (0–1) (n, %)	5 (41.7)	15 (23.4)	0.172 (0.035–0.907)	4.305	0.029
Symptomatic cerebral hemorrhage	1 (8.3)	3 (4.6)	0.351 (0.153–0.708)	0.518	0.610
90-d mortality (n, %)	1 (8.3)	6 (9.4)	0.785 (0.339–1.354)	1.462	0.227

OR: odds ratio; CI: confidence interval; mRS: modified Rankin scale.

## Discussion

In this study, 1,694 AIS patients arrived at the hospital emergency department within 4.5 h of the onset of stroke, and 76 (4.5%) patients had a previous history of cerebral hemorrhage. Based on the contraindication in stroke guidelines, those 76 patients should not receive intravenous thrombolysis, regardless of the time interval of cerebral hemorrhage occurrence, bleeding site, bleeding volume, and recovery condition of cerebral hemorrhage. According to their family's wishes, for some patients who had a cerebral hemorrhage long before and an optimal recovery, tPA intravenous thrombolysis was used at a median tPA dose of 0.64 mg/kg (*vs* 0.79 P=0.003 for patients without a history of cerebral hemorrhage). For safety, patients with a history of cerebral hemorrhage were treated with a low dose of tPA (0.6 mg/kg) thrombolytic therapy. A significant difference was observed among patients with a history of hypertension (83.3 *vs* 62.0%, P=0.028) and drinking (50 *vs* 30.5%, P=0.039) in the two groups. Hypertension may be the most important risk factor for cerebral hemorrhage, and the interaction between hypertension and alcohol in stroke is complicated (12–14). No significant difference was found in other baseline characteristics.

No significant difference in the 90-d mRS score of 0–1 (41.7 *vs* 43.6%, P=0.530) and the 90-d mortality rate (8.3 *vs* 6.5%, P=0.946) was noted, suggesting that both groups experienced similar treatment effects, without an increase in the mortality rate. However, a significant difference in SICH (8.3 *vs* 4.3%, P=0.039) between the groups suggests a higher risk of bleeding in patients with a history of cerebral hemorrhage after treatment with tPA intravenous thrombolysis, although this could be a result of bias due to the small number of patients with a previous history of cerebral hemorrhage who received thrombolytic therapy in this study. The mortality rate did not increase in patients after tPA intravenous thrombolysis, implying that they may benefit from this therapy.

Lee et al. (15) performed a retrospective analysis of 1,495 cases of intravenous thrombolysis, 73 of which included a history of cerebral hemorrhage (4.9%); incidence of SICH was 6.8 *vs* 4.6% in patients with or without a history of cerebral hemorrhage after thrombolysis, respectively, and no significant difference was found between them. AbdelRazek et al. (16) suggested that patients with a history of cerebral hemorrhage who received thrombolytic therapy had no increased risk of SICH. This conclusion was based on a retrospective analysis of brain imaging data from 640 patients admitted to a hospital between January 2006 and April 2014, who received tPA intravenous thrombolysis and who had a history of cerebral hemorrhage prior to receiving thrombolysis. Twenty-seven of these patients had experienced previous cerebral hemorrhage and only 1 (3.7%) patient presented SICH after tPA intravenous thrombolysis. Twenty-five cases (4.1%) presented SICH among the remaining 613 patients who received thrombolytic therapy. Meretoja et al. (17) studied 985 AIS patients who were eligible for treatment with alteplase but received thrombolytic therapy: 1 patient had a previous a history of cerebral hemorrhage and 2 patients had a history of subarachnoid hemorrhage. No SICH occurred after thrombolysis, although 2 patients had 90-d mRS scores of 0–1, and 1 patient had an mRS score of 3. These results suggest that a history of cerebral hemorrhage is not an absolute contraindication for thrombolytic therapy and that tPA intravenous thrombolysis does not increase the incidence of SICH and mortality rates among recipients.

The current study found a significant difference among patients with a previous history of cerebral hemorrhage who received tPA thrombolytic therapy and conventional therapy in terms of 90-d mRS scores of 0–1 (41.7 *vs* 23.4%, P=0.029, respectively), while no significant difference was found in incidence of SICH (8.3 *vs* 4.6%, P=0.610, respectively) or 90-d mortality (8.3 *vs* 9.4%, P=0.227, respectively). These findings demonstrate that tPA intravenous thrombolysis provides a better prognosis than conventional treatment for patients with a previous history of cerebral hemorrhage, with no significant difference in SICH, mortality, and safety. Based on safety considerations, when administering thrombolytic therapy, physicians should take into account patient history, particularly the length of cerebral hemorrhage and the recovery condition. Choosing patients who previously experienced better recovery leads to better mRS scores of 0–1 (91.7 *vs* 78.1%, P=0.022) and who have no medical history of cerebral hemorrhage (6.5 *vs* 3 years, P=0.003) results in significant differences among patients treated with thrombolytic therapy and those with conventional therapy. This indicates that tPA intravenous thrombolysis is safe and effective for some AIS patients with previous cerebral hemorrhage of a long duration but who had experienced positive recovery.

Taking into account the potential risk of SICH after thrombolysis, all AIS patients with a history of cerebral hemorrhage should be given low doses of intravenous thrombolysis (median tPA dose = 0.6 mg/kg). Japan Alteplase Clinical Trial (J-ACT) research results suggest that a low dose of tPA (0.6 mg/kg) intravenous thrombolytic therapy in the Japanese population can obtain similar clinical efficacy as the standard dose of National Institute of Neurological Disorders and Stroke (NINDS) and can reduce the risk of SICH (18). Although the J-ACT study did not establish a control group, the subsequent J-ACT II (19) and J-MARS studies (20) showed the safety and efficacy of intravenous thrombolytic therapy with low-dose tPA (0.6 mg/kg). The results of enhanced control in a hypertension and thrombolysis stroke study showed that low-dose tPA led to a lower risk for intracranial hemorrhage compared to the standard dose (0.9 mg/kg) and that low dose tPA reduces mortality rates (21). Thus, low-dose tPA thrombolysis is suggested to reduce the risk for intracranial hemorrhage among patients at higher risk for the condition when treated with thrombolysis.

This study was limited by being retrospective rather than prospective. Additionally, the number of patients included in the two treatment groups was not adequately balanced, especially for patients with a history of cerebral hemorrhage who were treated with intravenous thrombolytic therapy, which may have affected the results. Patients with this history were not randomly divided, and as a result, patients at a low risk for bleeding received intravenous thrombolytic therapy, which might also have affected the results. Finally, because cerebral hemorrhage occurred long before among patients treated with intravenous thrombolysis, imaging data had been lost; thus, bleeding volume was unclear. Thus, the relationship between amount of bleeding and risk related to tPA intravenous thrombolysis could not be determined.

In summary, these results suggest that a history of cerebral hemorrhage is not an absolute contraindication for thrombolytic therapy. In some patients, tPA intravenous thrombolysis does not increase the risk of SICH or mortality, and these patients may benefit from intravenous thrombolysis. Prospective, randomized controlled trials with large sample sizes from multiple treatment centers are needed to observe the relationship between incidence of cerebral hemorrhage and the amount of bleeding and the safety of thrombolysis.
